# Quality Evaluation of *Potentilla fruticosa* L. by High Performance Liquid Chromatography Fingerprinting Associated with Chemometric Methods

**DOI:** 10.1371/journal.pone.0149197

**Published:** 2016-02-18

**Authors:** Wei Liu, Dongmei Wang, Jianjun Liu, Dengwu Li, Dongxue Yin

**Affiliations:** 1 College of Forestry, Northwest A&F University, Yangling, China; 2 Agricultural College, Henan University of Science and Technology, Luoyang, China; 3 College of Landscape Architecture and Arts, Northwest A&F University, Yangling, China; 4 College of Agricultural Engineering, Henan University of Science and Technology, Luoyang, China; The University of Melbourne, AUSTRALIA

## Abstract

The present study was performed to assess the quality of *Potentilla fruticosa* L. sampled from distinct regions of China using high performance liquid chromatography (HPLC) fingerprinting coupled with a suite of chemometric methods. For this quantitative analysis, the main active phytochemical compositions and the antioxidant activity in *P*. *fruticosa* were also investigated. Considering the high percentages and antioxidant activities of phytochemicals, *P*. *fruticosa* samples from Kangding, Sichuan were selected as the most valuable raw materials. Similarity analysis (SA) of HPLC fingerprints, hierarchical cluster analysis (HCA), principle component analysis (PCA), and discriminant analysis (DA) were further employed to provide accurate classification and quality estimates of *P*. *fruticosa*. Two principal components (PCs) were collected by PCA. PC1 separated samples from Kangding, Sichuan, capturing 57.64% of the variance, whereas PC2 contributed to further separation, capturing 18.97% of the variance. Two kinds of discriminant functions with a 100% discrimination ratio were constructed. The results strongly supported the conclusion that the eight samples from different regions were clustered into three major groups, corresponding with their morphological classification, for which HPLC analysis confirmed the considerable variation in phytochemical compositions and that *P*. *fruticosa* samples from Kangding, Sichuan were of high quality. The results of SA, HCA, PCA, and DA were in agreement and performed well for the quality assessment of *P*. *fruticosa*. Consequently, HPLC fingerprinting coupled with chemometric techniques provides a highly flexible and reliable method for the quality evaluation of traditional Chinese medicines.

## Introduction

Traditional Chinese medicine (TCM), with a history of five thousand years, is still in extensive demand [1]. TCM possesses a significant position in the Chinese health system [2] and is being closely examined for the development of novel pharmaceuticals [3,4]. However, challenges for the guarantee of quality of TCM accumulate gradually. There has always been resistance to the adoption of TCM worldwide because of its complexity, the presence of unknown components and the lack of quality control. Chromatographic fingerprint analysis of key components is regarded as a reasonable approach for the quality evaluation of complicated TCMs [5]. A chromatographic fingerprint displays an average chromatographic map of all the samples, rather than a chromatogram of an individual sample [6]. This differs from conventional practice in which one or more components are chosen as active markers for detection and quality evaluation. Fingerprinting relies on the intrinsic relationships between multiple components and generates a defining pattern for TCM samples. Chromatographic fingerprinting, a more important technique for insuring the quality of Chinese medicines and their products, has been acknowledged extensively by many countries and organizations [7,8] such as the European Medicines Agency, the US Food and Drug Administration, the British Herbal Medicine Association and the Indian Drug Manufacturers’ Association [5]. To facilitate the quality assessment for vast and varied TCMs, the World Health Organization (WHO) has accepted chromatographic fingerprinting as a technique for the evaluation of medicinal species [7–10]. Hence, chromatographic fingerprinting has received increasing attention recently among accessible quality control techniques.

Chemometric methods, especially cluster analysis and principle component analysis (PCA), which collect chemical profiles objectively, have been widely applied for assortment and identification in analyzing the chemical information of herbal medicines [11,12]. Furthermore, HPLC techniques coupled with multivariate statistical methods (chemometric methods) have been employed extensively to classify and distinguish various herbs [8,13–17].

*Potentilla fruticosa* L., a member of the Rosaceae family, is commonly used as a natural tea and also as an important TCM [18,19]. Apart from its common applications as a garden plant, a food additive and in cosmetics [20,21], the plant, commonly known as the “Jinlaomei drug” in China, has numerous medicinal virtues for strengthening the stomach and the spleen, promoting metabolism, regulating menstruation and for relieving feelings of tiredness [19,22]. Modern academic studies have revealed that the medical foundations of *P*. *fruticosa* are related to various chemical ingredients [23–27] that have strong antioxidant activity [28–30] and that are contained in the leaves. *P*. *fruticosa* is native to North America and is widely distributed in the Qinghai, Gansu, Ningxia, Sichuan, Yunnan and Tibet regions in China [19]. For this widespread species, the active ingredients may vary, thus the contents, properties and proportions of the constituents vary because of the distribution in different geological zones. This will cause the same species from different growing regions to possess different therapeutic properties, making the quality assessment of *P*. *fruticosa* extremely crucial. Additionally, because applications for *P*. *fruticosa* are growing consistently, exploring a reliable quality assessment method is critical.

Chemical and pharmacological research on *P*. *fruticosa* has led to the discovery of several types of bioactive components, such as flavonoids and phenolics [23–24]. In previous studies [25], quercetin and kaempferol were generally regarded as markers for investigating the active ingredients of *P*. *fruticosa* by HPLC. Nevertheless, this is far from adequate because there must be more than these two compounds contributing to the general efficacy and pharmacological activities of *P*. *fruticosa*. A comprehensive investigation of *P*. *fruticosa* based on the chemical fingerprint has not been reported. Moreover, objective methods that are able to assess the similitude among HPLC fingerprints and distinguish the disparities between samples are in urgent demand. Therefore, the HPLC fingerprint method coupled with multivariate statistical techniques may be applied to perform a quality evaluation of *P*. *fruticosa*.

This paper is designed to introduce a more available HPLC fingerprint technique that can better reveal and evaluate the properties of *P*. *fruticosa*. The main active components (tannins, flavonoids, total phenolics, rutin, quercetin and kaempferol) and antioxidant activities were quantified, and the HPLC fingerprint method was explored and verified using eight samples that were collected from its representative areas which covered all of distribution zones of China. Similarity analysis (SA) [31], hierarchical cluster analysis (HCA) [16,17,32–34], principle component analysis (PCA) [35,36] and discriminant analysis (DA) [8,13–17] were applied to the HPLC fingerprint profiles to obtain a more direct figure for the evaluation of the similarities and distinctions between the tested samples. The combination of fingerprint analysis and chemometric techniques is expected to provide a significant and powerful approach for conducting a comprehensive quality assessment of *P*. *fruticosa* in the future.

## Materials and Methods

### Instrumentation and reagents

HPLC analyses were conducted on an Agilent Series 1260 liquid chromatograph at ambient temperature. The instrument is comprised of a quaternary gradient pump, a variable wavelength detector system and a reversed-phase SB-C18 column (5 μm, 4.6×250 mm, Agilent, USA). Data acquisition was performed with ChemStation software (Agilent, USA).

Folin-Ciocalteus’s phenol reagent was purchased from Solarbio Co., Ltd (Beijing, China). 2,4,6-Tripyridyl-s-triazine (TPTZ), 6-hydroxy-2,5,7,8-tetramethylchroman-2-carboxylic acid (Trolox), 2,2-azino-bis (3-ethyl-benzothiazoline-6-sulfonic acid) diammonium salt (ABTS) and 1,1-Diphenyl-2-picrylhydrazyl (DPPH) were purchased from the Sigma-Aldrich Co., St. Louis, USA. Standards including tannic acid, rutin, gallic acid, quercetin and kaempferol were obtained from the Chinese National Institute for the Control of Pharmaceutical and Biological Products (Beijing, China). Acetic acid, sodium nitrite, HPLC grade methanol, sodium hydroxide and sodium carbonate were provided by the Tianjin Bodi Chemical Holding Co. Ltd (Tianjin, China), who also supplied the other chemicals of analytical grade. Deionized water (18 MΩ cm) served as the solvent for aqueous solutions. Stock solutions of all reagents employed here were prepared in methanol and diluted to the required concentration.

### Plant materials

**Fig 1:**
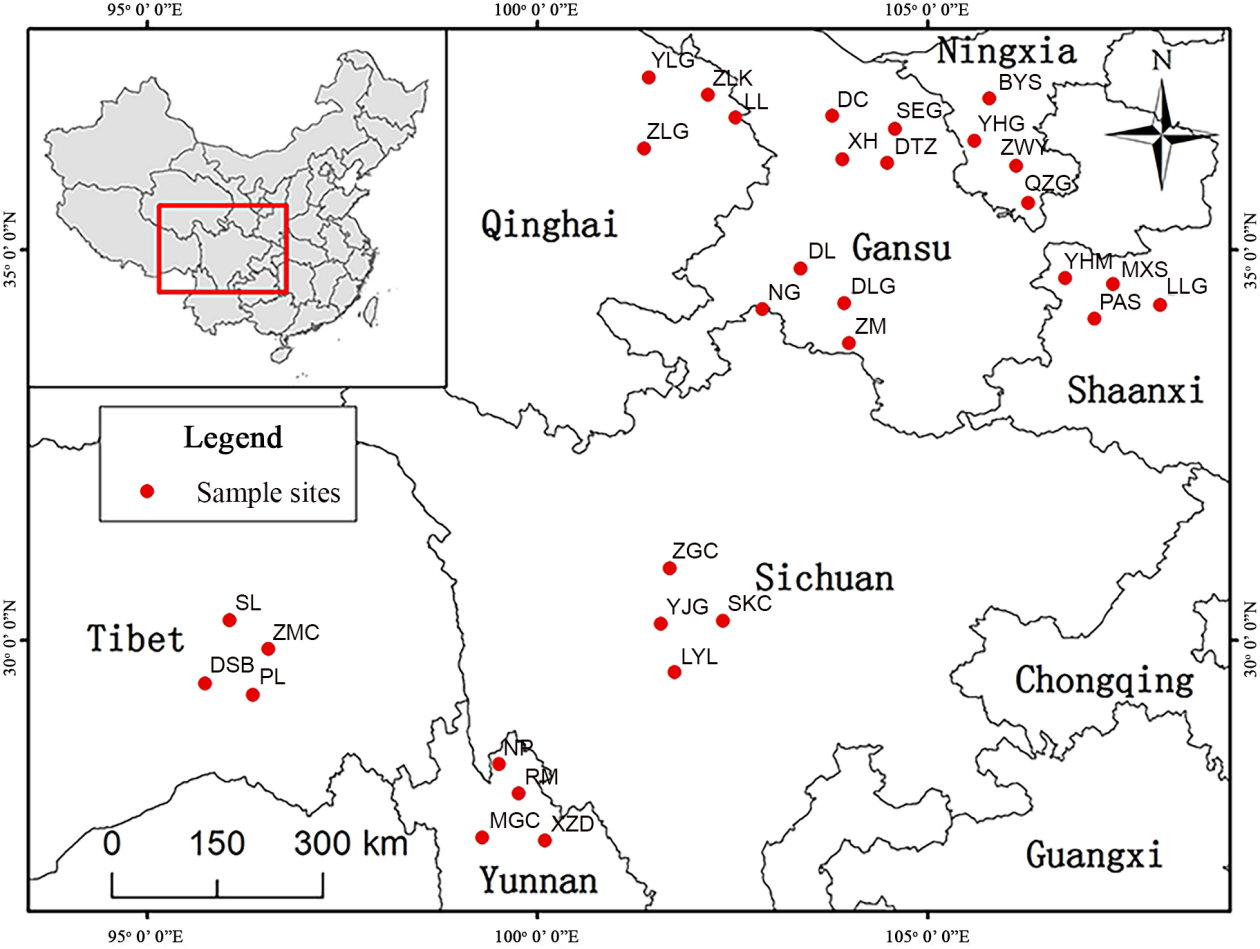
Locations of *P*. *fruticosa* samples in different regions sampled for this study. Maps generated using ArcGIS 10.0 (ESRI Inc. 2014).

*P*. *fruticosa* were collected from eight representative regions of China in July, 2014 (Fig 1). Specifically, a total of twenty healthy specimens from four populations (each population was separated geographically by at least 30 km, and 5 m for adjacent individuals) with similar growth stature were collected in each test region, as shown in Table 1. Mature leaves were harvested from four orientations (north, south, east and west) of different positions (upper, middle and lower parts) of each individual and then evenly combined as one test sample in each test region [2], thereby obtaining 8 test samples. All samples were dried at 40°C under a vacuum. After grinding into powders, the samples were stored in the dark at −20°C before further use. Voucher specimens from all populations were identified by Professor Jianjun Liu of Northwest A&F University, and were deposited at the Herbarium of Northwest A&F University (WUK0780381-0780388).

**Table 1:** *P*. *fruticosa* samples collected from different regions of China.

No.	Locations	Population	Code	Coordinates	Number of samples	Altitude (m)	Group
S1	Mei county, Shaanxi	Pingansi	PAS	E107°43′N34°1′	5	2815	B
		Mingxingsi	MXS	E107°44′N34°0′	5	2637	
		Yuhuangmiao	YHM	E107°22′N34°5′	5	1780	
		Liulingou	LLG	E108°10′N33°52′	5	1013	
S2	Diebu, Gansu	Zemo	ZM	E103°21′N33°45′	5	2728	B
		Dalong	DL	E103°14′N35°2′	5	2620	
		Dalagou	DLG	E103°22′N33°52′	5	2677	
		Nagai	NG	E103°14′N33°51′	5	2963	
S3	Huzhu, Qinghai	Zhalongkou	ZLK	E102°34′N36°53′	5	2264	C
		Zhalonggou	ZLG	E102°37′N36°47′	5	2698	
		Yuanlongogu	YLG	E102°27′N36°54′	5	3069	
		Lalagou	LL	E102°42′N36°44′	5	3169	
S4	Jingyuan, Ningxia	Baiyunshan	BYS	E106°15′N35°37′	5	2232	C
		Yehegu	YHG	E106°13′N35°31′	5	2370	
		Zhiwuyuan	ZWY	E106°18′N35°22′	5	2080	
		Qiaozigou	QZG	E106°22′N35°15′	5	2564	
S5	Yongdeng, Gansu	Suoergou	SEG	E102°43′N36°40′	5	2389	C
		Xiahe	XH	E102°43′N36°35′	5	2733	
		Dachang	DC	E102°44′N36°44′	5	2449	
		Datanzigou	DTZ	E102°46′N36°33′	5	2530	
S6	Shangri-la, Yunnan	Rime	RM	E99°37′N27°51′	5	3528	C
		Naipi	NP	E99°36′N28°2′	5	3432	
		Xiaozhongdian	XZD	E99°56′N27°28′	5	3590	
		Mugaocun	MGC	E99°34′N27°30′	5	2250	
S7	Nyingchi, Tibet	Zhangmaicun	ZMC	E94°20′N29°40′	5	3097	C
		Selong	SL	E94°11′N29°44′	5	3173	
		Pula	PL	E94°22′N29°27′	5	3256	
		Duosongba	DSB	E94°13′N29°37′	5	3855	
S8	Kangding, Sichuan	Yajaigeng	YJG	E101°57′N30°0′	5	2946	A
		Laoyulin	LYL	E101°59′N29°55′	5	3788	
		Shengkangcun	SKC	E102°1′N30°4′	5	3207	
		Zhonggucun	ZGC	E101°54′N30°16′	5	3554	

### Ethics statement

No specific permissions were requested for any locations and activities, such as the field sampling studies described above. No areas privately owned and species on the verge of extinction or protected were involved during the collection of plants and relevant studies.

### Preparation of the extracts

Considering the impact of various factors, the extraction process was optimized using a response surface method [37]. The optimized extraction process was as follows: extraction time, 2 h; ethanol concentration, 70%; extraction temperature, 45°C; number of extractions, three; liquid-solid ratio, 15:1. Each powdered sample was treated as described in the optimized extraction process. The filtrates that were obtained were evaporated at 40°C under a vacuum using a rotary evaporator, and were then stored in the dark at 4°C for later use. If necessary, the extracts were diluted. All extractions were conducted in triplicate.

### Measurement of tannin

Tannin contents were determined using the Folin-Denis method [38]. First, 1.0 mL of diluted sample solution (2 mg mL^−1^) was transferred to a 25 mL volumetric flask to which was added 1 mL of F-D chromogenic reagent and 5 mL of sodium carbonate solution (1 mol L^−1^). The solution was diluted to 25 mL by the addition of methanol. After incubating for 30 min at room temperature, the absorbance at 720 nm was measured against a blank. Tannic acid (1–10 mg L^−1^) was used to construct a standard curve. All measurements were conducted in triplicate.

### Measurement of total flavonoids

The total flavonoid contents were obtained using the sodium nitrite-aluminum nitrate colorimetric method [39]. Sample solution (1.0 mL, 2 mg mL^−1^) was transferred to a 25 mL volumetric flask and 0.3 mL of NaNO_2_ (5%) was then added and the solution was allowed to stand for 6 min. Next, 0.3 mL of Al(NO_3_)_3_ (10%) was added and the solution was held for another 6 min. Finally, 4 mL of NaOH (1 mol L^−1^) was added and the solution was diluted to 25 mL with 70% ethanol. After incubating for 30 min at room temperature, the absorbance at 510 nm was measured against a blank. Rutin (4–40 mg L^−1^) was used to prepare the standard curve. All measurements were performed in triplicate.

### Measurement of total phenolics

The total phenolic contents were determined using a modified Folin-Ciocalteu colorimetric method [40]. Sample solution (1.0 mL, 2 mg mL^−1^) was transferred to a 25 mL calibration flask and 0.5 mL of Folin-Ciocalteu reagent and 2.5 mL of sodium carbonate (1 mol L^−1^) were added and the solution was diluted to a final volume of 25 mL with methanol. After 30 min of incubation at 30°C, the solution was centrifuged at 4,000 rpm (10 min) and the absorbance at 760 nm was measured against a reagent blank. Gallic acid (0–6 mg L^−1^) was used to prepare a standard curve. All measurements were conducted three times for each sample.

### RP-HPLC (reversed-phase high performance liquid chromatography) analysis

Prior to analysis, sample solutions (1 mg mL^−1^) were filtered through 0.22 μm membrane filters and were then separated by RP-HPLC [41–45]. The contents of three monomeric chemical substances (quercetin, rutin and kaempferol) were also analyzed by RP-HPLC. Water with 0.5% acetic acid (mobile phase A) and methanol with 0.5% acetic acid (mobile phase B) formed the mobile phases. The flow rate was 1 mL min^−1^. The gradient program was as follows: 0–15 min, 25% to 35% B; 15–30 min, 35% to 60% B; 30–40 min, 60% to 100% B; 40–45 min, hold B at 100%. The injection volume and detection wavelength were 20 μL and 360 nm, respectively. Analyses were performed in triplicate.

### DPPH radical scavenging assay

For the evaluation of antioxidant activity in *P*. *fruticosa* leaves from different locations, a modified DPPH technique was employed to obtain the radical scavenging properties of different samples [26,41,46,47]. Samples to be tested (1–100 μg mL^−1^) and positive controls (rutin, 1–70 μg mL^−1^) were prepared with methanol, then 2.0 mL of this solution was mixed with 2.0 mL of 0.1 mol L^−1^ DPPH in methanol. The mixture was stirred and kept in the dark for 30 min at room temperature. The absorbance of the solution was measured at 517 nm against a reference blank. All tests were performed in triplicate. DPPH free radical scavenging activity (SA) can be expressed with the following formula:

$$\text{SA}\left(\text{\%}\right)=[1-\left(A_{i}-A_{j}\right)/A_{0}]\times 100$$

where A_i_ and A_j_ are the absorbances of 2 mL of the sample solutions mixed with 2 mL of DPPH and methanol, respectively. A_0_ is the absorbance of 2 mL of methanol mixed with 2 mL of DPPH.

### Ferric reducing antioxidant power (FRAP) assay

In this reaction, the color changed from yellow to green, based on the reducing capacity of the sample. Fe^3+^/ferricyanide complex was reduced to the ferrous form by reductants in the solution. Thus, Fe^2+^ can be traced as the reducing power index by measuring the absorbance [48]. The FRAP study was conducted with modifications [49,50]. The FRAP reagent was prepared daily by incubating a mixture of 10 mL of ferric trichloride hexahydrate (20 mM) solution, 10 mL of TPTZ (10 mM in 40 mM hydrochloric acid) solution and 100 mL of 0.3 M acetate buffer (pH 3.6) at 37°C. For each analysis, 400 μL of sample (0.1 mM) were mixed with 3 mL of FRAP solution. The increase in the absorbance at 593 nm was monitored at 15 s intervals over 30 min at 37°C. The FRAP profiles were displayed as micromoles of Trolox equivalent per gram of dry samples (μmol equiv. Trolox g^−1^). All tests were conducted in triplicate.

### ABTS·^+^ radical cation decolorization assay

The decolorizing free radical ABTS·^+^ method was performed with some modifications to determine antioxidant activity [51]. The ABTS·^+^ is a mixture of an ABTS stock solution (7 mM in water) and 2.45 mM potassium persulfate. The ABTS·^+^ can be maintained for 12–16 h while protected from light at room temperature prior to use. The ABTS·^+^ solution was diluted to the desired concentration with phosphate buffered saline (PBS, pH 7.4) until the absorbance reached 0.700 (± 0.021) at 734 nm. The solution was prepared fresh prior to each analysis. For the spectrophotometric analysis, 100 μL of each sample (0.1 mM) was added to 3.9 mL of the ABTS·^+^ solution. The decrease in absorbance was recorded at 734 nm. The data was expressed as micromoles of Trolox equivalent per gram dry weight of sample (μmol equiv. Trolox g^−1^). All determinations were conducted in triplicate.

### Statistical analyses

As recommended by the Chinese Pharmacopoeia Committee, we employed Computer Aided Similarity Evaluation System (CASES) software to calculate the correlation coefficients of entire chromatographic patterns among samples, and to generate the simulated mean chromatograms as well as characteristic peaks. Based on the correlation coefficient (median), the software was also used to conduct the similarity analysis (SA) of different chromatograms [52]. The correlation coefficient was used to calculate the results unless otherwise noted. Hierarchical clustering analysis (HCA), principal component analysis (PCA) and discriminant analysis (DA) were performed using SPSS software (SPSS for Windows 19.0, SPSS Inc., USA) [5,17,53]. HCA was conducted to assort samples with regard to the similarities of their chemical properties. The method, average linkage between groups, and the cosine were employed in the measurements [16,17,31,36]. A score plot, demonstrating the chemical distinctions between chromatograms, and a loading plot that depends on the variation found in each variable [36] were provided by PCA, which was applied to assess similarities and distinctions among the samples. Through the use of discriminant functions that were generated from the known samples, DA offered the best identification and assortment among the groups [8,16,17]. DA further confirmed the results of the SA of HPLC fingerprint methods coupled with HCA and PCA, making the quality evaluation of *P*. *fruticosa* more reasonable.

The profiles were presented as the mean value ± SD (standard deviation). The data were processed by one-way analysis of variance (ANOVA). By employing SPSS 19.0, Duncan’s multiple range measurement was conducted to evaluate the significance of the distinction between sample means (*P* < 0.05).

## Results and Discussion

### Antioxidant activity of *P*. *fruticosa* from different regions

#### DPPH radical scavenging activity

**Fig 2:**
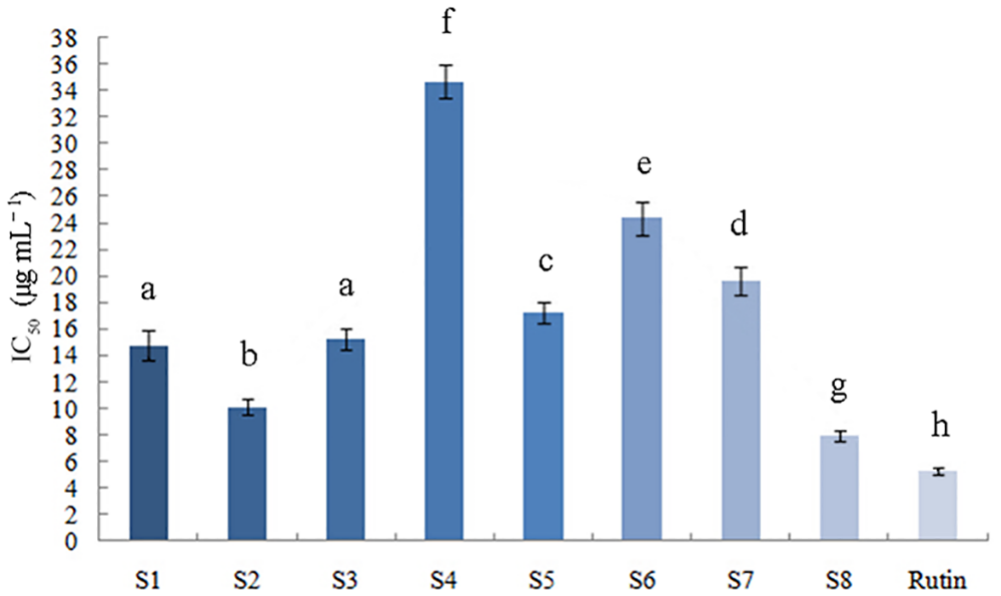
IC_50_ values of tested samples for DPPH radical scavenging activity.

The DPPH test has been applied extensively for the detection of antioxidant activity of pure antioxidant compounds and various plant extracts. IC_50_ values calculated from linear regression analyses are the effective concentrations under which DPPH radicals are eliminated by 50%, and a lower IC_50_ represents a stronger antioxidant capacity [46,47]. The DPPH radical scavenging capacity of the samples was tested and compared to assess the antioxidant capacity of *P*. *fruticosa*, and the IC_50_ values were presented in Fig 2. For *P*. *fruticosa* species collected from eight districts, the IC_50_ varied from 7.936 ± 0.423 to 34.68 ± 1.275 μg mL^−1^. The samples from Kangding, Sichuan (S8) possessed the highest DPPH radical scavenging activity with the lowest mean IC_50_ value of 7.936 ± 0.423 μg mL^−1^, which was similar to that of rutin standards (5.25 ± 0.265 μg mL^−1^). The DPPH radical scavenging capacity of the other two *P*. *fruticosa* samples collected from Diebu, Gansu (S2, IC_50_ = 10.115 ± 0.618 μg mL^−1^) and Mei county, Shaanxi (S1, IC_50_ = 14.763 ± 1.104 μg mL^−1^) were also high. We can infer that *P*. *fruticosa* could be a potential antioxidant natural resource. These profiles suggest that the antioxidant abilities of the same *P*. *fruticosa* species varied significantly from one growing location to another.

#### Ferric reducing activity power (FRAP) assay

**Fig 3:**
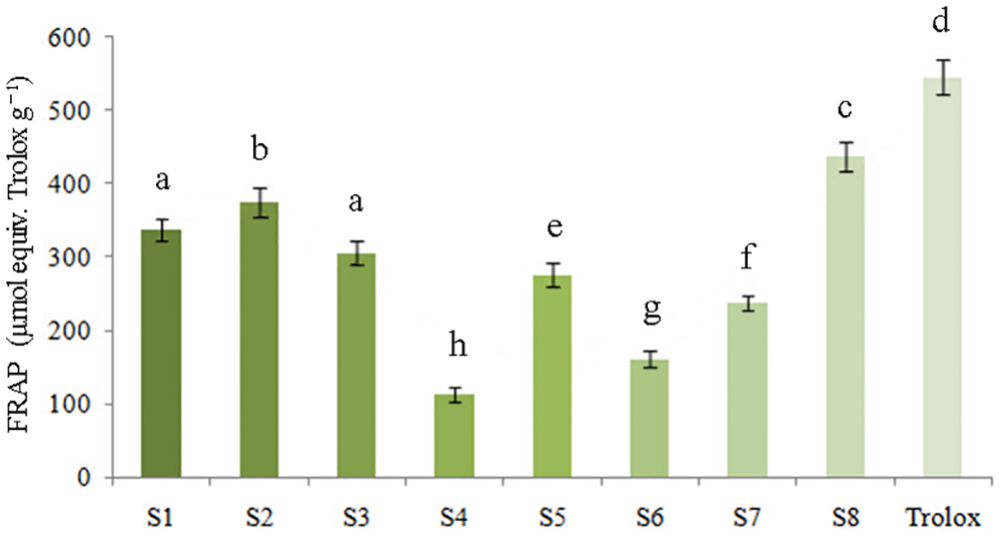
Reducing power of tested samples for ferric reducing activity power (FRAP) assay.

The FRAP analysis assessed the antioxidant activities of the *P*. *fruticosa* samples on the basis of their reducing ability, which was presented in Fig 3. As the *P*. *fruticosa* was sampled from eight locations, the FRAP values were greatly different (*P* < 0.05), and ranged from 112.24 ± 9.36 to 436.58 ± 20.62 μmol equiv. Trolox g^−1^. Specifically, *P*. *fruticosa* from Kangding, Sichuan (S8) possessed the highest FRAP value of 436.58 ± 20.62 μmol equiv. Trolox g^−1^ as the antioxidant capacity, followed by samples from Diebu, Gansu (S2) and Mei county, Shaanxi (S1) with values of 374.43 ± 19.47 and 337.91 ± 14.41 μmol equiv. Trolox g^−1^, respectively.

#### ABTS·^+^ radical cation scavenging activity

**Fig 4:**
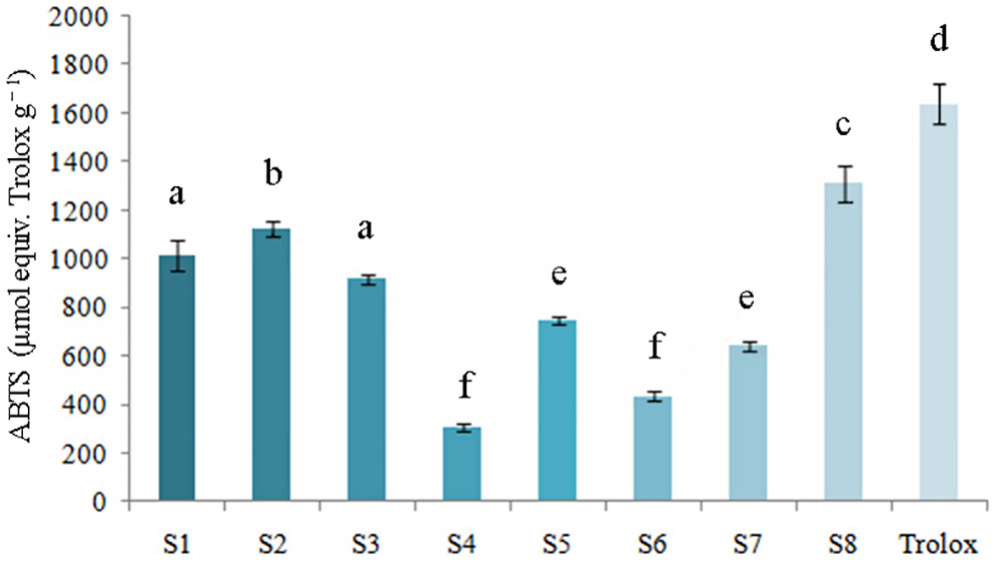
ABTS values of tested samples for ABTS·^+^ radical scavenging activity.

ABTS activity was determined on the basis of the percentage of ABTS·^+^ radical cation inhibited by antioxidants in the samples investigated. The ABTS profiles of the analyzed samples were shown in Fig 4, which is consistent with the FRAP values (Fig 3). Every sample exhibited an ability to neutralize the radical cation ABTS·^+^, and significant results (*P* < 0.05) were obtained. For *P*. *fruticosa* species from eight regions, the ABTS values were in the range of 303.048 ± 15.67 to 1309.74 ± 75.25 μmol equiv. Trolox g^−1^. The highest activity was detected in the samples from Kangding, Sichuan (S8) with a maximum value of 1309.74 ± 75.25 μmol equiv. Trolox g^−1^, followed by species from Diebu, Gansu (S2) and Mei county, Shaanxi (S1), with values of 1123.29 ± 34.33 and 1013.73 ± 65.12 μmol equiv. Trolox g^−1^, respectively.

The values of FRAP and ABTS displayed the same order as the antioxidant capacity detected by the DPPH method: S8>S2>S1>S3>S5>S7>S6>S4. These results consistently demonstrated that *P*. *fruticosa* exhibited both a high capability for free radical scavenging and for reduction. Additionally, the antioxidant ability of *P*. *fruticosa* species varied significantly among the same species from different growing regions (*P* < 0.05). Going forward, we would conduct further studies in cells and living bodies to fully reflect the antioxidant properties of *P*. *fruticosa*, which could not be achieved by the DPPH scavenging capacity assay, FRAP assay and ABTS·^+^ radical cation scavenging assay carried out above.

### Validation of the HPLC procedure

The linear range of the standards was obtained using a battery of the rutin, quercetin and kaempferol standard solutions. The detector response was linearly correlated with the concentrations of the standards: 1.0–100.0 μg mL^−1^ for rutin, 10.0–500.0 μg mL^−1^ for quercetin and 5.0–500.0 μg mL^−1^ for kaempferol. By plotting the peak areas (y) against the corresponding concentrations (x, μg mL^−1^), standard curves were generated, and the linearity of each was determined. The regression equations are y = 59142.31x−135.4 (r = 0.9998) for rutin, y = 2143.57x+ 1.6 (r = 0.9999) for quercetin and y = 24726.38x−12.86 (r = 0.9997) for kaempferol (Table 2).

**Table 2:** Method validation for the quantitative determination of three compounds using RP-HPLC.

Peak No.	Compounds	Regression equations	Test range (μg mL^-1^)	LOD (ng mL^-1^)	LOQ (ng mL^-1^)	Precision experiment (n = 7)		Repeatability experiment (n = 6)		Recovery experiment (n = 6)	
						Area of peak	RSD (%)	Area of peak	RSD (%)	Average recovery rate (%)	RSD (%)
2	Rutin	y = 59142.31x−135.4 R^2^ = 0.9998	1.0–100.0	2.89	9.98	1125.26	1.56	185.23	1.94	99.85± 0.02	1.72
3	quercetin	y = 2143.57x+ 1.6 R^2^ = 0.9999	10.0–500.0	1.87	8.14	2957.79	2.93	278.59	2.72	102.91± 0.01	2.23
8	kaempferol	y = 24726.38x−12.86 R^2^ = 0.9997	5.0–500.0	3.04	10.33	386.93	0.23	24.43	1.31	96.36± 0.02	1.16

LOD—Limit of Detection, LOQ—Limit of Quantitation, RSD—Relative Standard Deviation.

Each values represented in table are means ± SD (n = 7 for precision, n = 6 for repeatability and recovery experiment, respectively.).

Three compounds were identified by their relative retention time (RRT) (min): rutin (6.24, peak 2), quercetin (11.68, peak 3), kaempferol (31.57, peak 8).

**Fig 5:**
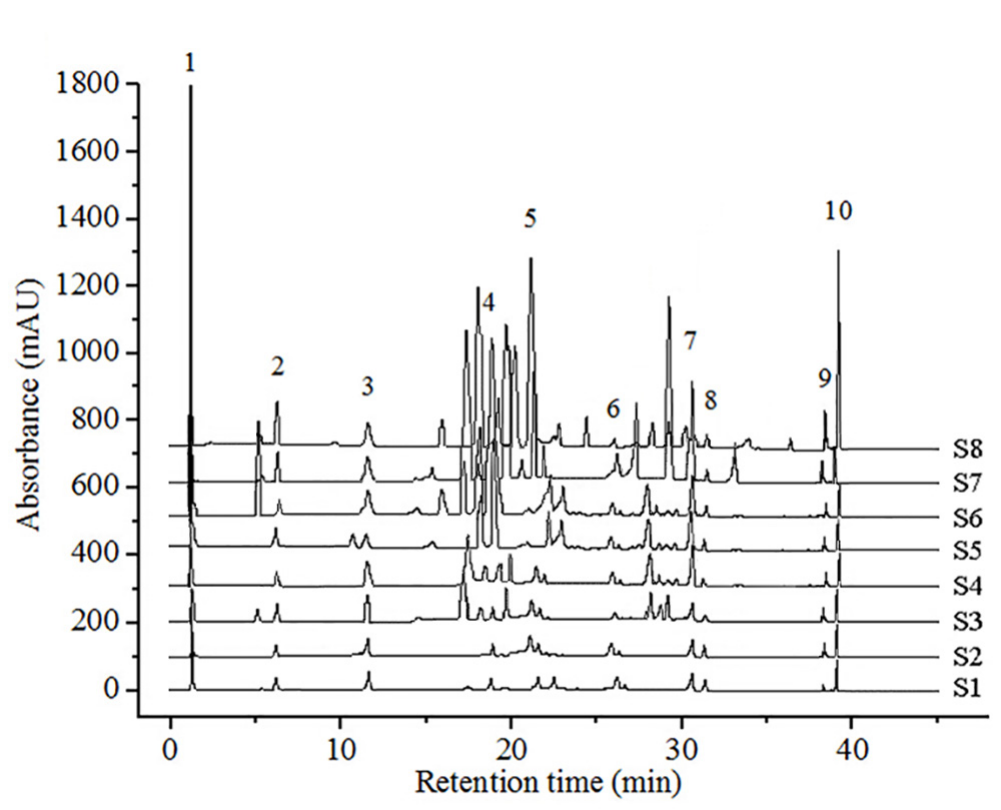
The HPLC fingerprinting profiles of *P*. *fruticosa* from different regions.

The precision and repeatability of the method were assessed using seven injections of sample solutions and six replicates of solid analytes, respectively [36]. For the peaks 2, 3, 8 (Fig 5) from duplicate injections, the precision of relative retention time (RRT) and relative peak areas (RPA) were found to be in the range of 0.02–0.07% and 0.23–2.93% of relative standard deviations (RSD) (n = 7), respectively. The RSDs of RRT and RPA of peaks 2, 3, 8 were calculated, respectively, to be 0.04–0.12% and 1.31–2.72% for the solid sample replicates (n = 6). The limit of detection (LOD) (signal/noise = 3) and the limit of quantification (LOQ) (signal/noise = 10) of the three compounds varied within the range 1.87–3.04 ng mL^-1^ and 8.14–10.33 ng mL^-1^.

By estimating the RPAs of the samples that were maintained for 0–24 h, the stabilities of peaks 2, 3, and 8 (Fig 5) were assessed, respectively. The RSDs of RRTs and RPAs were found to be less than 3%. To confirm the accuracy of the method, we conducted a recovery experiment in which quantified analytes were mixed with specific amounts of standard components. The average percent recoveries for the three peaks were in the range of 96.36 ± 0.02% to 102.91 ± 0.01%. The RSDs shifted from 1.16% to 2.23% (n = 6). These results suggest that the factors for the fingerprint analysis were optimal (Table 2).

### Differences in active ingredient contents

**Fig 6:**
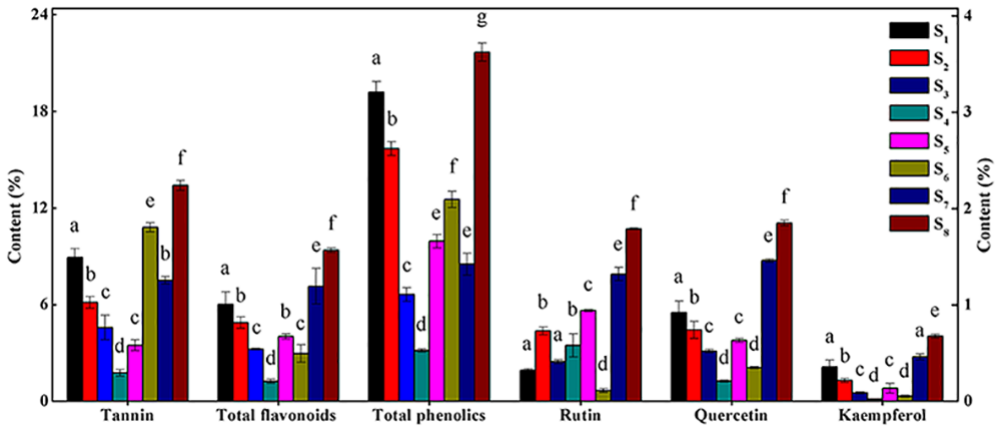
Differences of active ingredient contents in *P*. *fruticosa* samples from different regions. S1: Mei county, Shaanxi; S2: Diebu, Gansu; S3: Huzhu, Qinghai; S4: Jingyuan, Ningxia; S5: Yongdeng, Gansu; S6: Shangri-la, Yunnan; S7: Ningchi, Tibet; S8: Kangding, Sichuan.

For each *P*. *fruticosa* sample from the eight districts, the contents of tannin, total flavonoids and total phenolics varied greatly (Fig 6). The concentrations of these phytochemicals ranged from 1.762 ± 0.21 to 13.401 ± 0.32%, 1.24 ± 0.14 to 9.37 ± 0.36% and 3.14 ± 0.11 to 21.68 ± 0.57%, respectively. The contents of total phenolics were relatively high in all analytes, particularly in *P*. *fruticosa* samples from Kanding, Sichuan (S8). The contents of tannin and total flavonoids were most abundant in S8, whereas these substances were found at lower concentrations in *P*. *fruticosa* samples from Jinagyuan, Ningxia (S4). Li *et al*. also discovered that the contents of total flavonoids in *P*. *fruticosa* leaves from different environments displayed great differences, which agreed with the present results [22].

The contents of rutin (peak 2), quercetin (peak 3) and kaempferol (peak 8) in *P*. *fruticosa* from different regions were identified and calculated by a comparison of the RRTs and RPAs with those of the standards (Figs 5 and 6). Among all the constituents, the concentrations of the three identified compounds were at relatively low levels, but exhibited great differences. The contents of rutin, quercetin and kaempferol varied within the ranges of 0.11 ± 0.01 to 1.79 ± 0.01%, 0.21 ± 0.01 to 1.85 ± 0.03% and 0.022 ± 0.003 to 0.676 ± 0.02%, respectively. *P*. *fruticosa* from Nyingchi, Tibet (S7) had higher contents of these three compounds, and *P*. *fruticosa* from Kanding, Sichuan (S8) exhibited the highest contents among all samples. Rutin was especially low in *P*. *fruticosa* from Shangri-la, Yunnan (S6), whereas quercetin and kaempferol were especially low in samples from Jingyuan, Ningxia (S4). Chen *et al*. reported that the quercetin and kaempferol contents of *P*. *fruticosa* from distinct districts varied significantly [25], which further confirmed our findings. Referring to these findings, the amounts of active ingredients varied greatly with the geographical sources of *P*. *fruticosa* samples. These differences may be due to ecological factors, genetics, and the status of the secondary metabolism of *P*. *fruticosa*.

Owing to the antioxidant activity and potential protective health effects, flavonoids, the most common and widely distributed natural compounds, are of value in nutrition and medicine [54,55]. Recent studies have shown that environmental conditions play an important role in affecting the percentage of total flavonoids in plants. For instance, Bai *et al*. [30] and Li *et al*. [22] found that altitude had a significant positive effect on the total flavonoid contents in *P*. *fruticosa*. Annual average temperatures and altitude positively contributed to the contents of flavonoids in *Eucommia ulmoides* (*P* < 0.05). The amounts of flavonoids in Arabidopsis increased after long term illumination [56]. For this study it was known that the altitude, environmental temperature and illumination in Kangding, Sichuan are higher than in other locations, based on field investigation data. Additionally, *P*. *fruticosa* is a heliad; therefore, regions with large amounts of sunshine could be beneficial for its growth, leading to the generation of abundant substrates that can form secondary metabolites. This could provide an explanation for why *P*. *fruticosa* from Kangding, Sichuan had high amounts of total flavonoids.

Tannin, rutin, quercetin and kaempferol are common antioxidants that are beneficial for human health [57–59]. The results obtained in this study demonstrated that *P*. *fruticosa* contained significant amounts of these active phytochemicals, and may be a promising source of antioxidants for the food and drug industries.

In summary, in terms of the elaborate analyses and assessments, *P*. *fruticosa* samples collected from Kangding, Sichuan (S8) were selected as the most valuable raw materials due to their having the highest contents of combined phytochemicals (tannin, total flavonoids, total phenolics and three identified compounds) and the strongest antioxidant activity (IC_50_ = 7.936 ± 0.423 μg mL^−1^, FRAP value = 436.58 ± 20.62 μmol equiv. Trolox g^−1^ and ABTS value = 1309.74 ± 75.25 μmol equiv. Trolox g^−1^). Furthermore, Kangding, Sichuan was the district most beneficial for the growth of *P*. *fruticosa*. By contract, Jingyuan, Ningxia was considered an unsuitable region because the lowest contents of active ingredients (except for rutin) and antioxidant activities were observed in samples from that region (S4).

### Similarity analysis (SA) of HPLC fingerprints

Eight *P*. *fruticosa* samples that were collected from distinct areas were investigated to define a standard fingerprint using the developed HPLC conditions. Ten common peaks were selected as characteristic peaks, and peak 3 (quercetin), at a retention time of 11.68 min (Fig 5), was selected as the reference standard peak. By analyzing the chromatograms (Fig 5), species with the same origin from different areas could tentatively be identified. Additionally, we applied the CASES software to assess the similarity of the chromatograms, and found that the correlation coefficients were in the range of 0.21 to 0.92 (Table 3). A high degree of similarity was detected between Qinghai (S3) and Gansu samples (S5), which were usually regarded as the same medicinal herb with similar chemical constituents. Detection of this similarity suggested the disparities of different samples, indicating that the chromatograms were characterized and related to phytochemical compositions [36].

**Table 3:** Similarities of the chromatograms of *P*. *fruticosa* samples based on the correlation coefficients.

No.	S1	S2	S3	S4	S5	S6	S7	S8
S1	1.00							
S2	0.67	1.00						
S3	0.63	0.63	1.00					
S4	0.72	0.58	0.81	1.00				
S5	0.54	0.72	0.92	0.88	1.00			
S6	0.74	0.33	0.37	0.67	0.71	1.00		
S7	0.37	0.51	0.52	0.42	0.83	0.61	1.00	
S8	0.29	0.43	0.21	0.53	0.71	0.32	0.62	1.00

### Hierarchical clustering analysis (HCA)

**Fig 7:**
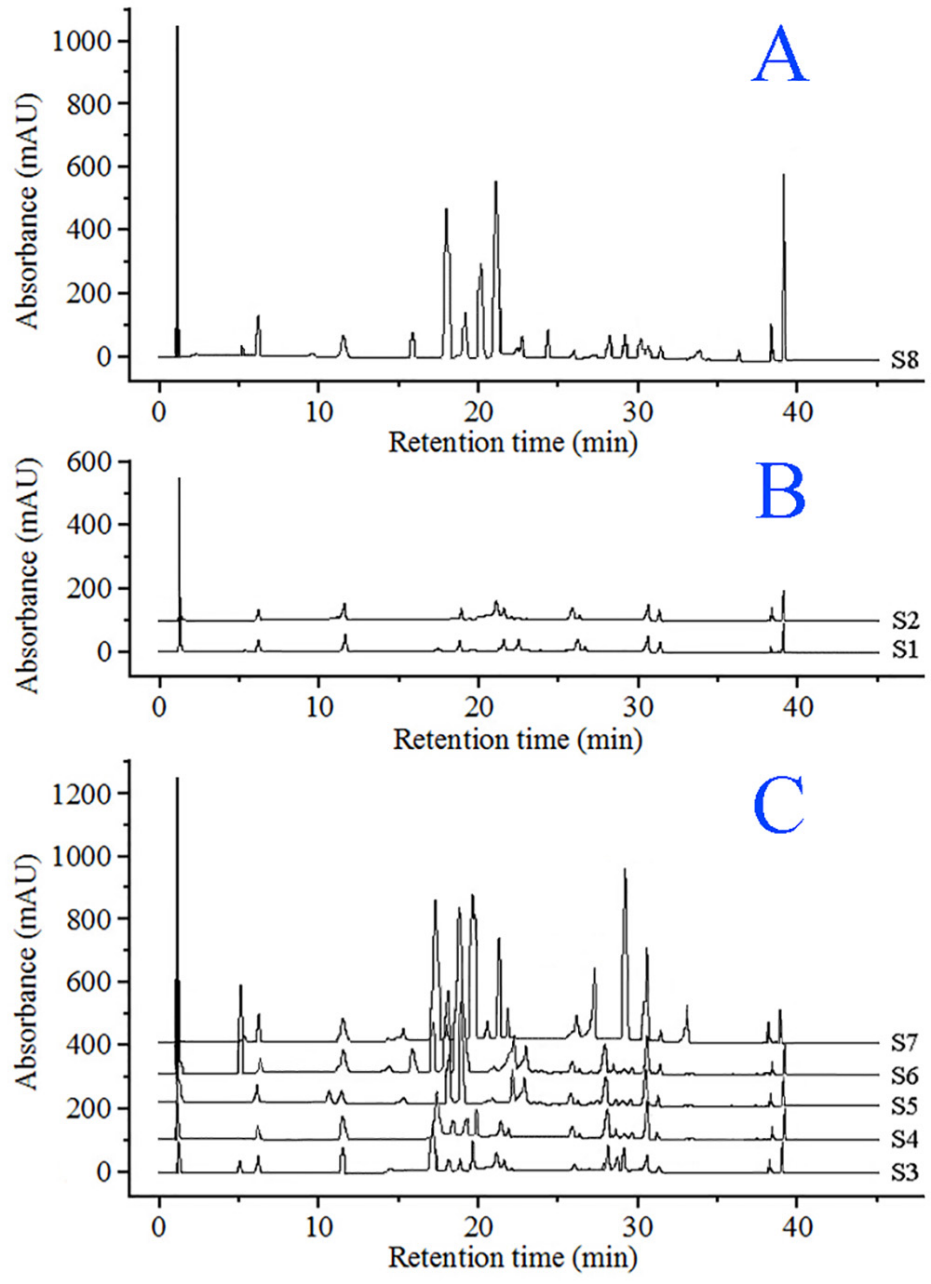
Visual assortment for HPLC chromatograms of *P*. *fruticosa* species.

**Fig 8:**
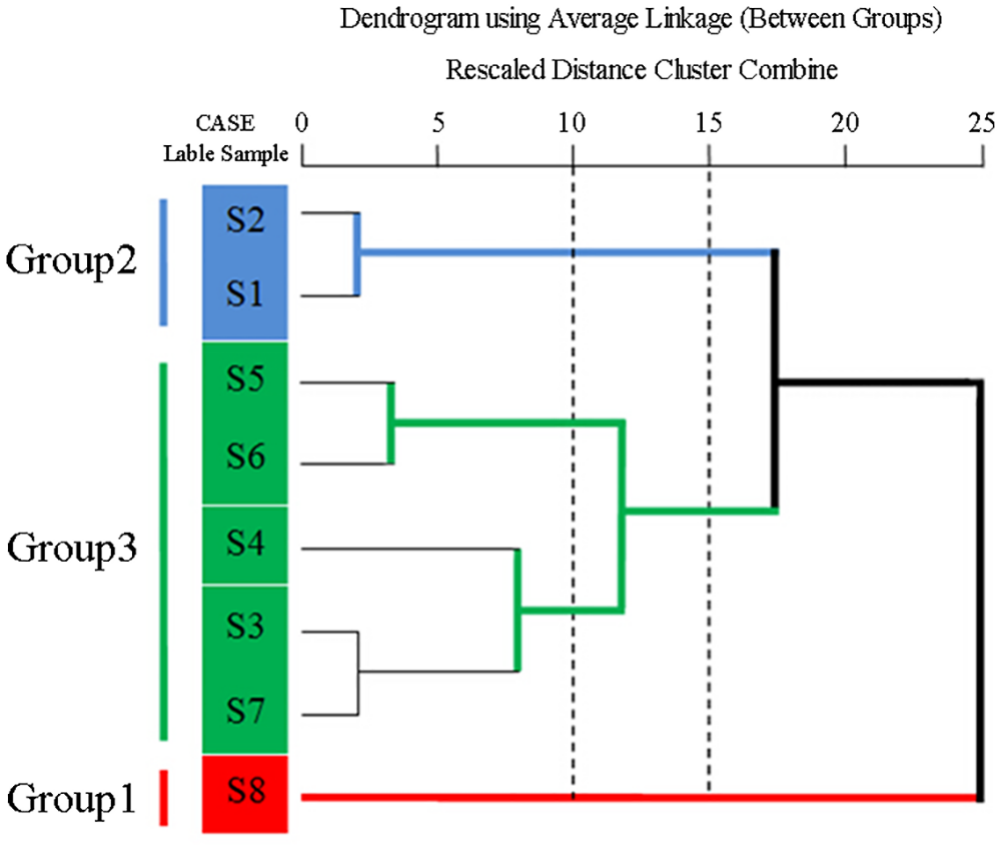
Dendrograms of hierarchical cluster analysis (HCA) for the analytes.

Sample data were analyzed visually by overlapping tests using the ChemStation software (Agilent, USA) and were divided into three distinct patterns A, B and C (Fig 7) before HCA. Visual comparisons of chromatograms are subjective and non-quantitative. Hence, an HCA was applied to process the fingerprint data (RPAs of the 10 common chromatographic peaks) of *P*. *fruticosa* samples to assess quantitatively the similarities and distinctions of chromatographic peaks [17,31]. A 10×8 matrix was formed by the RPAs of common ingredients in 8 batches of *P*. *fruticosa* species from different sources. Using the average linkage between groups and the cosine method, we obtained an HCA-dendrogram (Fig 8), in which the quality properties were clearly displayed. Assuming that a proper level of distance (15) had been selected, the analytes would be assorted into three quality clusters, which revealed the subjectivity of visual classification. Group 1 was formed only by the samples collected from the Sichuan province (S8). Group 2 included the samples collected from the Shaanxi province (S1) and Gansu province (S2). Owing to the similarity of the chemical constituents, the other five samples, S3, S4, S5, S6, and S7 were clustered in group 3. For the contents of active ingredients and antioxidant activity, it can be seen that group 1 had the highest contents of tannin, total flavonoids, total phenolics, rutin, quercetin and kaempferol, and the highest antioxidant activity. The different qualities of samples from different production locations were most likely due to factors such as the altitude, latitude and longitude of these locations, along with soil nutrients.

The correlation coefficients of chromatograms within groups 1, 2 and 3 corresponding to the simulated mean chromatograms generated from the software, and the correlation coefficients between the simulated mean chromatograms were displayed in Table 4. Within a particular group, the chromatograms were almost constant. According to the simulated profiles, chromatograms were sorted into a particular group, for which the correlation coefficients were more than 0.95. Nevertheless, the chromatograms within a particular group were significantly distinct from that of other groups, and the distinctions in the similarity values for the three groups (Table 4) corresponded to the data generated from HCA. The correlation coefficients between the simulated mean chromatograms were less than 0.60, and ANOVA investigations indicated that the distinctions between different groups were significant (*P* = 0.041). These results demonstrated that HCA can distinguish *P*. *fruticosa* species with the same origin from different districts, which confirmed divisions based on morphological characterizations (Table 1).

**Table 4:** Correlation coefficients between individual chromatograms within a group and the group simulative mean chromatogram, and between the group simulative mean chromatograms.

Group	Group1	Group2	Group3
Group1	1.000 a (n = 1)	0.561 b	0.534 b
Group2		0.954±0.005 a (n = 2)	0.585 b
Group3			0.971±0.000 a (n = 5)

^a^ Correlation coefficient of individual chromatograms to the simulative mean chromatogram of the corresponding group. Values are the mean ± SD.

^b^ Correlation coefficient between simulative mean chromatograms.

### Principal component analysis (PCA)

**Fig 9:**
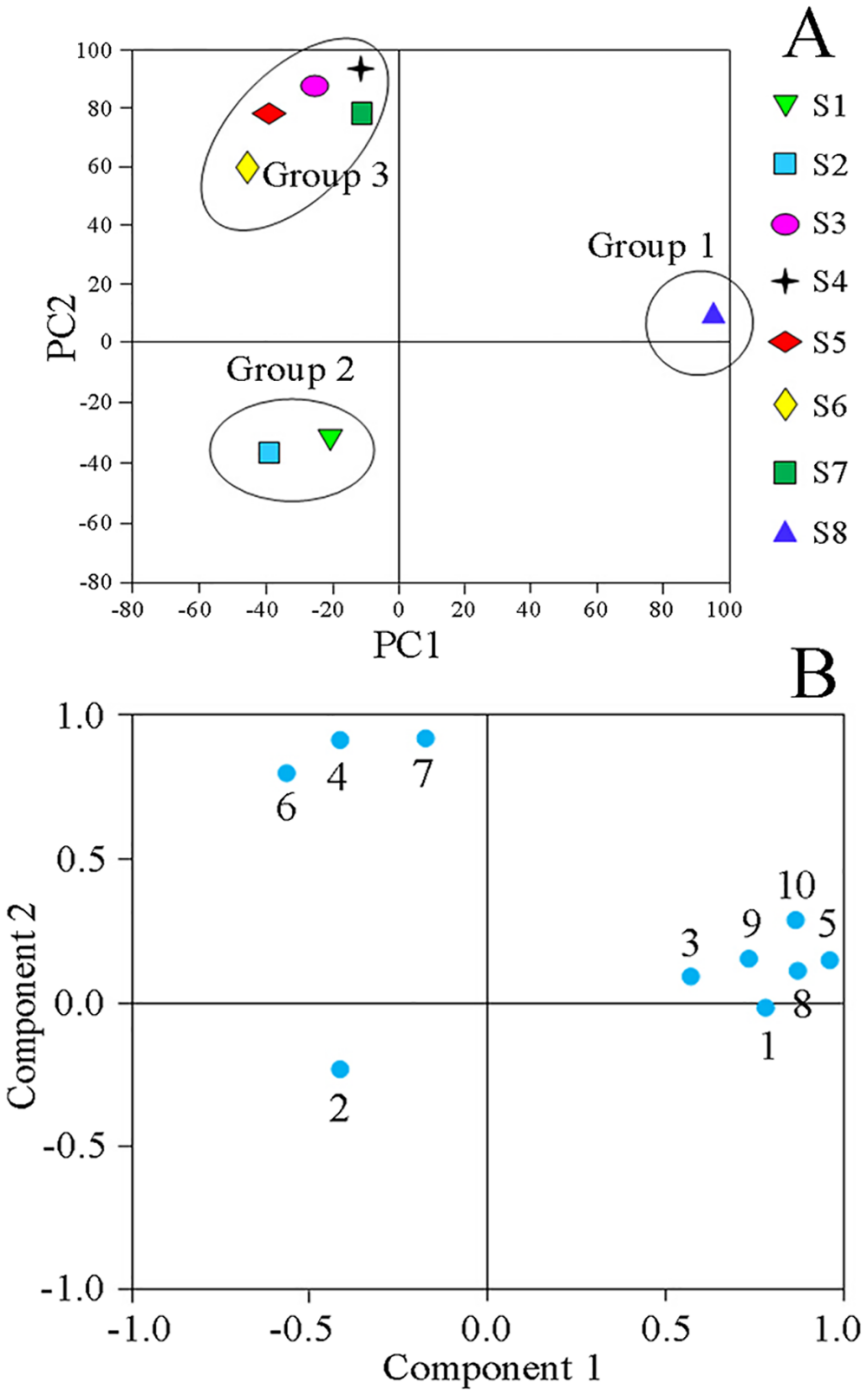
The scores plot generated from principal component analysis (PCA) of all the analytes (A) and the loadings plot of variables (peaks 1–10) (B).

From the chromatograms of samples investigated (Fig 5), we could infer that the content disparities of common compounds contributed most to the differences among samples. To determine the discrimination capacity of these common constituents, PCA was conducted using the RPAs of common peaks, as with HCA as input data. The variability in the original observations for the first and second PCs was 57.64% and 18.97%, respectively. Thus the total variances of the first two PCs were estimated to be 76.61%. Therefore, the multidimensional information could be concentrated into a 2-D dataset to assort the samples. Examination of the scores plot (Fig 9A) that was acquired by analyzing the HPLC data of 8 samples, revealed a positive influence on the quality evaluation of *P*. *fruticosa*. The scores sorted the analytes into three large groups, labeled as groups 1, 2, and 3, which were relevant to the different chemical profiles due to the distinctions of the locations. PC1 distinguished S8 (group 1) from other samples while PC2 discriminated S3 ~ S7 (group 3). The rest of the samples composed group 2. Analysis of the loading plots of PC1 against PC2 (Fig 9B) revealed that peaks explained by PC1, 1, 3, 5, 8, 9 and 10, had an influence on the cluster in a top-down order. These were the vital compounds that distinguished S8 from other samples. By comparison, peaks 4, 6 and 7, that mainly contributed to PC2, distinguished S3 ~ S7 from other samples. Thus it was easy to determine the different chemical components and properties of each group through the scores and loadings plots.

Due to the higher intensities of peaks 1, 3, 5, 8, 9 and 10, group 1, composed of *P*. *fruticosa* samples from Kangding Sichuan (S8), was characterized primarily by positive values of PC1. By contrast, owing to the relatively lower intensities of peaks 1, 3, 5, 8, 9 and 10, group 2, containing samples from Mei county, Shaanxi (S1) and Diebu, Gansu (S2), was separated by negative values of PC1. Because of the high intensities of peaks 4, 6 and 7, group 3 (S3 ~ S7) was characterized by positive values of PC2. These results can offer a good explanation for the HCA results.

In summary, because more peaks were explained by PC1 than PC2, we could imply that the samples on the right side of Fig 9A, were of higher quality than the others. If there is more than one sample, the sample with the higher space is considered to be better [36]. Hence, we could draw the conclusion that *P*. *fruticosa* collected from Kangding Sichuan (S8) possessed the highest quality.

### Discriminant analysis (DA)

On the basis of the characteristics obtained in each case, DA can be used to establish a predictive model for the group membership. Based on linear combinations of the predictor variables, which could discern the optimal discrimination among the groups, one or more discriminant functions for more than two groups could be developed from the course. The functions established from the samples with known membership are also applicable to new cases with the unknown ones [17]. Ten peaks were chosen from the patterns, thereby ten variables were built. However, not all these variables built contributed to the development of the discriminant function. By adopting only the useful ones, the DA would establish discriminant functions. Two kinds of discriminant functions were acquired using the SPSS software.

Canonical discriminant function:

$$Y_{1}=2.28X_{3}-5.152X_{4}+26.491X_{7}-2.346$$

$$\;\;Y_{2}=0.725X_{3}+3.554X_{4}+32.341X_{7}-5.718$$

Discrimination standard:

$$Y_{1}>0\;and\;Y_{1}>-Y_{2}:\;G_{1}$$

$$Y_{1}<0\;and\;Y_{1}<Y_{2}:\;G_{2}$$

$$Y_{2}<0\;and\;Y_{1}<-Y_{2}:\;G_{3}$$

Fisher’s discrimination function:

$$G_{1}=12.716X_{3}-8.227X_{4}+202.437X_{7}-26.743$$

$$G_{2}=-0.13X_{3}+33.741X_{4}+9.786X_{7}-22.325$$

$$G_{3}=1.886X_{3}-2.156X_{4}+44.233X_{7}-2.378$$

Standard for discriminant: according to the highest of the three functional values, each sample is assigned to the group. X represents the variable and G1, G2 and G3 denote the samples from groups 1, 2 and 3, respectively.

**Fig 10:**
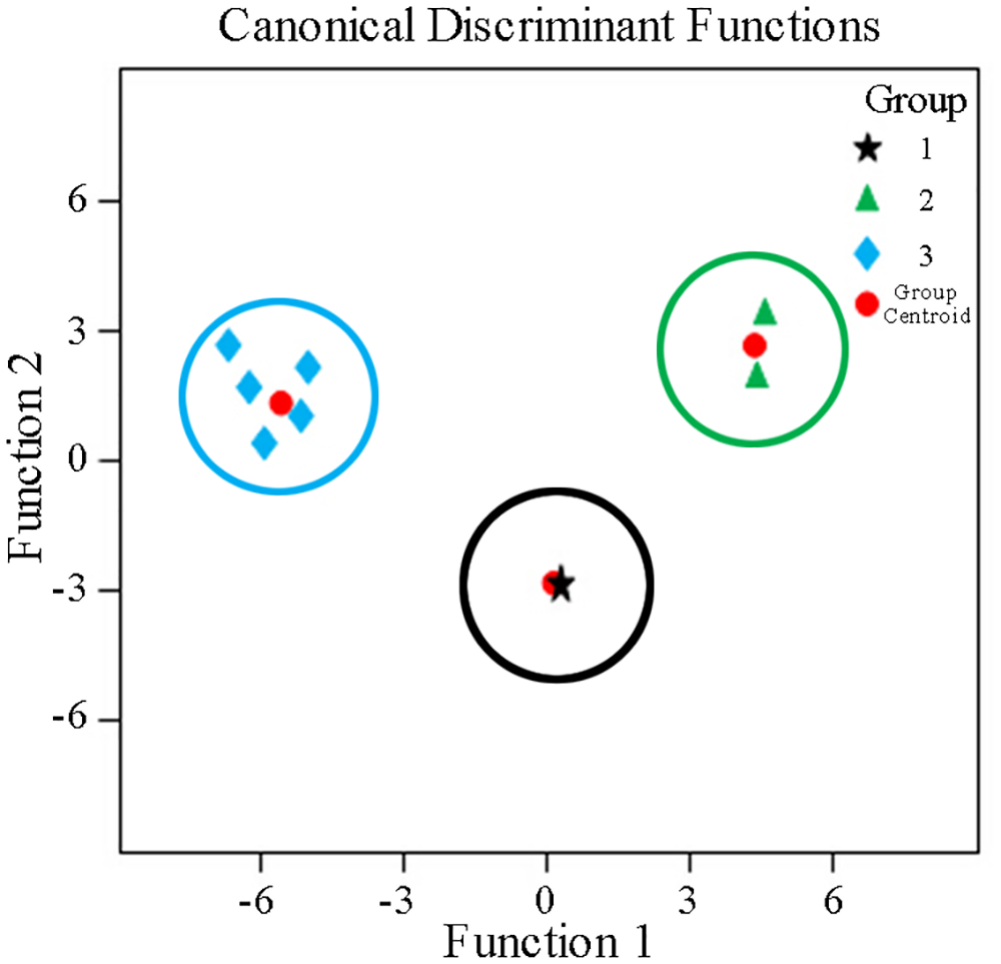
Discrimination analysis (DA) for *P*. *fruticosa* samples.

Only the variables assigned to the areas of peaks 3, 4 and 7 were adopted to develop the discriminant functions. By inputting values of the three variables into the formula, a discriminant standard value was obtained to group an unknown sample. The DA plots of high resolution for the three groups were displayed in Fig 10. By employing the three variables that were most discriminating, the analytes, included in groups 1, 2 and 3 could be divided with 100% accuracy.

Based on the HPLC fingerprint results, the chemical constituents of *P*. *fruticosa* species changed significantly along with the geographical sources. These rich variations in chemical compositions may explain the remarkable disparity in their quality as medicines, nutritional supplements and functional foods. Thus, it is necessary to assess the quality of *P*. *fruticosa* from different production locations. The results of HPLC fingerprint SA, HCA, PCA and DA in our study were in agreement, and the techniques performed well for the quality assessment of *P*. *fruticosa*.

The present research was also significant for the collection and application of *P*. *fruticosa* in medical care and food industries. It is well known that the quality of medical herbs reflects the integrated influences of composite factors during growth, such as environmental factors, genetic factors, cultivation methods and collection periods. Additionally, for widespread plant species, a variation of the chemical components within the same plant species may occur because of the widespread geological zones, resulting in quality differences. *P*. *fruticosa* is one such widespread plant species that is widely distributed in Asia, Europe and North America. It occurs widely in China, and eight growing locations were investigated in this research. Further research should include the collection of additional samples from other regions in China and other countries, which should be analyzed by HPLC fingerprinting combined with chemometrics and the examination of environmental and genetic factors.

## Conclusions

The results obtained from our study suggested that all of the *P*. *fruticosa* samples investigated possessed very distinct phytochemical patterns and antioxidant activities. Integrated comparison and analysis revealed that the *P*. *fruticosa* samples from Kangding, Sichuan (S8) stood out from the other samples because of its highest active ingredients and strongest antioxidant ability. From the view of the contents of active ingredients and antioxidant activity, Kangding, Sichuan province proved to be the most beneficial for the growth *P*. *fruticosa*. The combinations of RP-HPLC fingerprint SA, HCA, PCA and DA were further adopted to develop an effective method for quality assessment of *P*. *fruticosa*. This study showed that RP-HPLC fingerprints that contained accurate chromatographic data could better reflect the chemical compositions of species and appropriately demonstrated the quality characteristics of *P*. *fruticosa*. The matching and discrimination of fingerprints of an assortment of samples becomes a promising approach for sample comparison and quality assessment. Therefore, fingerprint analysis coupled with chemometric paths is an innovative, reliable and facile method that can provide a powerful and meaningful way to elaborately perform quality assessment of TCMs. Furthermore, although our research involves only *P*. *fruticosa*, the methodology, concentrating on discriminating the chemical compositions in species by proper modifications, possesses potential applications in species authentication and quality evaluation of other edible and medicinal plants.
